# Real-Time Weather Monitoring and Prediction Using City Buses and Machine Learning

**DOI:** 10.3390/s20185173

**Published:** 2020-09-10

**Authors:** Zi-Qi Huang, Ying-Chih Chen, Chih-Yu Wen

**Affiliations:** 1 Department of Electrical Engineering, Innovation and Development Center of Sustainable Agriculture (IDCSA), National Chung Hsing University, Taichung 40227, Taiwan; g107093014@mail.nchu.edu.tw; 2 Information Technology Department, Pou Chen Corporation, Taichung 40764, Taiwan; nathan.chen@pouchen.com

**Keywords:** weather monitoring, weather prediction, bus systems, machine learning

## Abstract

Accurate weather data are important for planning our day-to-day activities. In order to monitor and predict weather information, a two-phase weather management system is proposed, which combines information processing, bus mobility, sensors, and deep learning technologies to provide real-time weather monitoring in buses and stations and achieve weather forecasts through predictive models. Based on the sensing measurements from buses, this work incorporates the strengths of local information processing and moving buses for increasing the measurement coverage and supplying new sensing data. In Phase I, given the weather sensing data, the long short-term memory (LSTM) model and the multilayer perceptron (MLP) model are trained and verified using the data of temperature, humidity, and air pressure of the test environment. In Phase II, the trained learning model is applied to predict the time series of weather information. In order to assess the system performance, we compare the predicted weather data with the actual sensing measurements from the Environment Protection Administration (EPA) and Central Weather Bureau (CWB) of Taichung observation station to evaluate the prediction accuracy. The results show that the proposed system has reliable performance at weather monitoring and a good forecast for one-day weather prediction via the trained models.

## 1. Introduction

Weather plays an important role in people’s lives. Through weather monitoring, data analysis and forecasting can be performed to provide useful weather information [1]. In terms of forecasting, since there are many factors that affect weather changes, it is challenging to predict the weather accurately [2]. Considering system operations and processing technologies, the existing systems for weather monitoring and prediction can be described from the system architecture and the information processing perspectives, respectively.

From the system architecture perspective, weather monitoring stations can be static or mobile. With the information provided by the fixed meteorological stations, there is some simulation software that uses numeric simulation to define the temperature in each grid [3]. The precision of the estimated calculus for each grid is proportional to the number of weather stations distributed over the city. In Lim et al. [4], the National Weather Sensor Grid (NWSG) system is designed to monitor weather information in real time over distributed areas in a city, where the weather stations are set in schools. In Sutar [5], a system is developed to enable the monitoring of weather parameters like temperature, humidity and light intensity. However, mobility issues and communication protocols are not considered.

**Figure 1:**
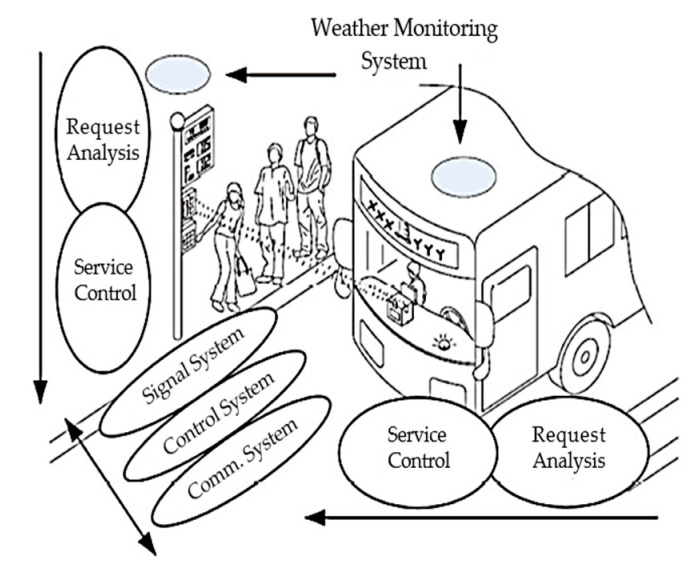
Intelligent bus system model for weather monitoring (reproduced from [9]).

The mobile weather station is installed on the vehicle, constantly driving in a specific area to collect data and send the data to different receivers via wired or wireless technologies, which leads to a better balance of coverage than static observatories. In Foina et al. [6], a city bus is applied as a mobile weather station to collect data through the path traveled by the vehicle. The system has three levels of interaction, the device integrated into the buses, terminal computer, and system computer. Although the PeWeMos system [6] argues that it may monitor the very fine details and weather changes within the one area and provide very fine weather information and changes in even a sufficient amount of time, the interpretation of the sensed weather data and the cooperation of the buses, bus stops, and passengers for weather monitoring are not addressed. Hellweg et al. [7] uses floating car data for road weather forecasts, which aims to increase the resolution of the weather observation network and the forecast model. The preliminary results show that bias corrections and quality control of the raw signals are key issues to enable safe autonomous driving. Considering the impact of communication channels on information quality, the weather’s impact on the performance of a radio link had been studied in [8], which analyzes the correlation between several weather variables and the behavior of control frames in an outdoor wireless local area network. Based on the bus information management system, our previous work [9] combines the advantages of local information processing and bus mobility, and proposes a real-time weather monitoring system, including a weather monitoring system and a management subsystem between buses and stations. Figure 1 shows the system model, including signal, control, and communication components.

From the information processing perspective, since forecasting is a very important analysis topic, machine learning provides a capability to the systems to learn and improve from experience [10,11]. Moreover, with machine learning, data analysis, and prediction can be achieved without understanding the physical processes (e.g., applying the past data to predict future data [12]). Readers may refer to [13] for a full discussion.

In the literature, many prediction models for rainfall and weather forecasting have been proposed. For instance, Parashar [14] proposes a system for monitoring and reporting weather conditions so as to be notified in advance to take relative measures to reduce possible damage. An Arduino Mega is used with some weather sensors to display the sensed values on the LCD screen, and the machine learning technology is applied to train the model and prediction and put the prediction results on the website. It mainly monitors the weather conditions and predicts the average, maximum and minimum temperature of the next day, not providing more detailed information like the weather conditions (e.g., temperature, humidity, and air pressure) for each hour.

Singh et al. [15] develops a low-cost, portable weather prediction system that can be used in remote areas, with data analysis and machine learning algorithms to predict weather conditions. The system architecture uses the Raspberry Pi as the main component with temperature, humidity, and barometric pressure sensors to obtain the sensed values and then train according to the random forest classification model, and predict whether it will rain. Note that although the system hardware in Singh et al. [15] and that of the proposed weather monitoring and forecasting systems are similar, the system in Singh et al. [15] only describes the probability of precipitation. In Varghese et al. [16], with Raspberry Pi and weather sensors, data are collected, trained, and predicted using linear regression machine learning models for evaluation via mean absolute error and median absolute error.

Instead of only considering the information processing perspective, this work simultaneously adopts the system architecture and the information processing perspectives. On the basis of the system architecture in Chen et al. [9], a pair of bus stops and a bus, the gateway, and the server can work as a group to dynamically operate the control system and communication system, which extend the system to apply the collected data with machine learning algorithms for providing weather monitoring and forecasting. Note that given basic meteorological elements such as pressure, temperature, and humidity, this work focuses on the prediction of the temperature, humidity, and pressure for the next 24 h with mild weather changes. For the forecast of severe weathers, in order to accelerate the training process and improve the predictive accuracy, Zhou et al. [17] state the predictors should contain major environmental conditions, which include meteorological elements such as pressure, temperature, geopotential height, humidity, and wind, as well as a number of convective physical parameters (i.e., including additional and advanced sensor equipment) to build the prediction system.

The major contributions and features of this work are: (1) proposition of a novel real-time weather monitoring and prediction system with basic meteorological elements; (2) development of an information processing scheme for increasing the management efficiency via a bus information system; (3) construction of machine learning models to analyze the trend of weather changes and predict the weather for the next 24 h. Table 1 describes the performance comparison of existing and proposed systems, which shows that besides temperature prediction, the proposed system is able to provide a forecast of basic meteorological elements (e.g., temperature, humidity, pressure) for one-day weather prediction via the trained models.

The rest of the paper is organized as follows: Section 2 depicts the system architecture, including information processing, data transmission/reception processes, the system components, and the implementation of the system. Section 3 presents machine learning models and input data formats. Section 4 describes the experimental results of each processing block and depicts the performance comparison of different prediction models. Finally, summarize this research in Section 5.

## 2. System Description

### 2.1. Overview

**Figure 2:**
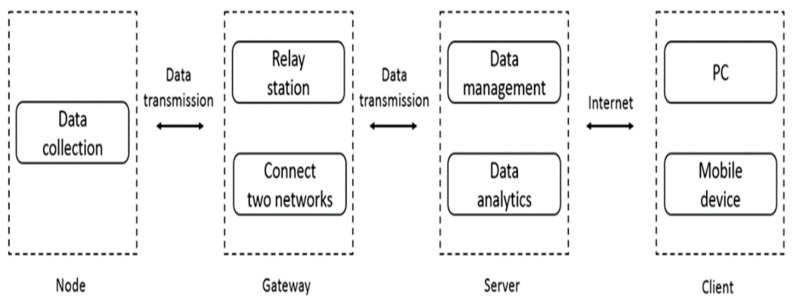
Overall system block diagram.

The proposed system is developed via an information management system, transmission technologies, signal processing, and machine learning technologies. Considering the operations among the system components (i.e., nodes, the gateway, the server, and the client), Figure 2 shows the overall system block diagram of communication and data transmission/reception. The functions of each system component are described as follows:

1. Nodes: The nodes are mainly for buses and bus stations to obtain weather information through the Raspberry Pi with sensors (e.g., temperature and humidity sensors, Near-Field Communication (NFC) modules, etc.). The nodes on a bus transmit the information to those on a neighboring bus station, and then the nodes on the bus station send the data to the gateway.
2. Gateway: The gateway is a relay station, used to upload the data to the server for the next processing step and connect two networks, one of which is composed of the nodes, the other uses the Internet Protocol Suite.
3. Server: The server is a data center and control platform at the backend of the website, which receives the data transmitted by the gateway, classifies the data, stores it in the database, and then applies the data for integrating machine learning prediction and model training. The server delivers the data requested by the site and responds to the client with the desired information. With the current and former weather information, the proposed system is able to perform temperature, humidity, and barometric pressure forecasts for the next 24 h (detailed in Section 3).
4. Client: Users can browse the website through PCs and mobile devices to obtain the current weather information or the forecasting for planning their daily activities.

### 2.2. System Architecture and Operations

**Figure 3:**
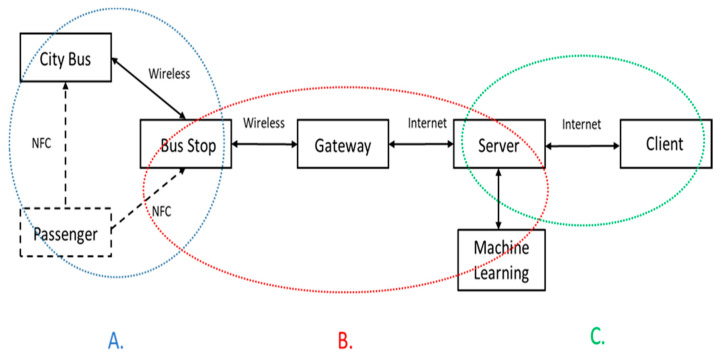
The system architecture: the dashed lines indicate the communication links related to passengers, and the solid lines indicate the communication links between each part of the system. Note that parts (**A**), (**B**), and (**C**) represent bus–station operations, station–gateway–server operations, and server–client operations, respectively.

Figure 3 shows the system architecture, composed of three major parts: Figure 3A bus–station operations, Figure 3B station–gateway–server operations, and Figure 3C server–client operations, which introduce the message flow between each part, including data collection, information sharing, data processing, and data storage.

#### 2.2.1. Bus–Bus Station Operations

##### Hardware/Software Implementation

In the bus–bus station block (i.e., block A), Raspberry Pi 3 Model B+ [18] is used on buses and stations with several weather sensors and wireless communication modules to achieve data collection and transmission. The hardware structures on the bus and the bus station include the Raspberry Pi and the sensors (i.e., temperature, humidity, barometric pressure, PM 2.5, ultraviolet, and raindrop sensors). Here we briefly explore the usage of wireless technologies for system operations. For modeling the interactions among the bus, the bus station, and passengers, the Near-Field Communication (NFC) module is applied to simulate the EasyCard system on a Taiwan bus to count the number of people and signal sensing at the bus station to request parking. The bus and the station use the wireless communication module XBee Serial 2 (S2C) for data transmission. The station also uses the LoRa module which is used for wireless transmission with the Gateway. Since Raspberries have only digital inputs, some sensors need to be paired with the MCP3008 to convert analog signals. The current weather information can also be viewed via the monitors of the bus and station. With the Raspberry Pi running on the Linux system, in the experiment, Python programming is used to obtain the sensing values and process the data, and a MySQL database is built on the Raspberry Pi of the bus and station to record the data.

##### Communication Operation

**Figure 4:**
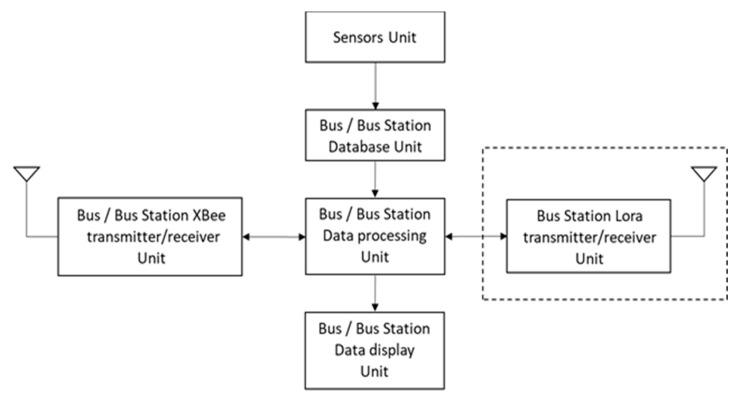
Framework on the bus and the bus station, where the bus station is with the dotted box.

Figure 4 (without the dashed box) shows the process of collecting, storing, and transmitting the sensor data of the bus. When the NFC module obtains the number of passengers or the measurements from weather sensors, the data are stored in the MySQL database. After the data are processed, they will be transmitted to the bus station via the XBee module, and then the user can view the weather information through the screen. At the bus station, the data sent from the bus through the XBee module are stored in the database, which will be forwarded to the gateway via LoRa wireless technology. Figure 4 (with the dashed box) shows the process of data transmission/reception at the bus station.

#### 2.2.2. Bus Station–Gateway–Server Operations

The LG01 LoRa gateway [19] is used as a relay station between the station and the server. The station transmits the data to the gateway, and then the gateway uploads the data to the server with the LoRa wireless technology, which bridges a LoRa wireless network to an IP network. For the software implementation, writing a C program in the Arduino development environment allows Gateway to receive signals sent by LoRa at the bus station, and then upload it to the server through the HTTP network protocol.

#### 2.2.3. Server–Client Operations

With the Windows 7 development environment and a Django network framework, Python and JavaScript are used for programming, and SQLite is used for setting the database. The software implementation of the proposed weather system includes a web server and a database in order to perform data processing, storage, and website display. When the data is recorded to the database, an Excel file will be exported at the same time, which contains the time, temperature, humidity, and air pressure values. Afterwards, Python in the Jupyter Notebook environment and the learning model are applied to make weather predictions and upload the results for storage, where the prediction information is shown on the website. Table 2 details all the sensor information in the database, including the time, the detection values, and the ID of a bus stop of five sensors (i.e., an ultraviolet sensor, a raindrop sensor, a temperature/humidity sensor, an air pressure sensor, and a PM 2.5 sensor). Note that the row of Pred temp is a set of predicted values of humidity, temperature, and pressure. Table 3. describes the measurement unit of each sensor.

### 2.3. Machine Learning

To build a prediction model, this work applies the opensource data of the Environmental Protection Administration (EPA) and the Central Weather Bureau (CWB) in Taiwan [20], about 50,000 hourly measurements in the last six years of the Taichung Observatory, as the training data source. Every measurement includes temperature, humidity, and air pressure at a 1 h measurement interval. Before training the model, first, process the dataset. Next, organize the data according to a specific format, and then perform predictive model training. Accordingly, the temperature, humidity, and air pressure values are first taken out from the opensource dataset. Next, the measurement data is divided into training dataset, test dataset, and validation dataset, and then the average and standard deviation of the three datasets are taken. Finally, the data are standardized.

## 3. Input Data Format

**Figure 5:**
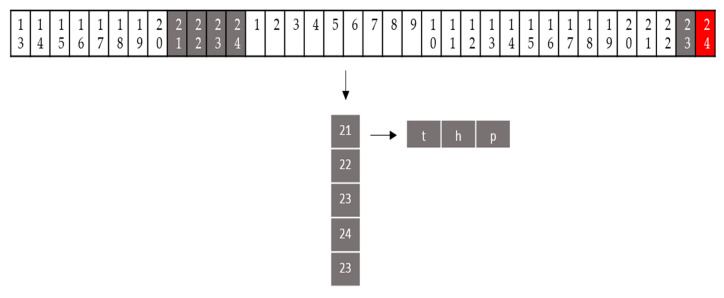
The first type of input data format (G1) represented by a timeline, where each data contains three values: temperature t, humidity h, and pressure p.

In order to examine the prediction performance, the measurement data is processed through different formats (i.e., G1, G2, G3, and G4) as shown in Table 4. Let $In_{T}^{D}$ indicate the sensing value at what time of the day, where In represents the input data, D indicates the present day, and T represents the time in 24 h format. Denote the day before as D-1. For instance, $In_{23}^{D}$ represents 23:00 of the present day and $In_{22}^{D-1}$ represents 22:00 of the previous day. Each type of input data format is represented by a timeline, where each data contains three values: temperature t, humidity h, and pressure *p*. Assuming that the weather data at 24:00 (i.e., the red part of Figure 5) is to be predicted, the G1 format is considered for the evaluation of adjacent time periods (i.e., the input data (the gray parts of Figure 5) will contain four entries from 21:00 to 24:00 yesterday and data from 23:00 today). The rationale for the G1 format is to explore the data characteristics at short adjacent time periods. For the G2, G3, and G4 formats, we investigate the impact of the data formats at moderate time period (e.g., G2 for the past 12 h) and long time period (e.g., G3 for the past 24 h and G4 for the past 48 h) on the prediction performance.

### Learning Model Architecture

**Figure 6:**
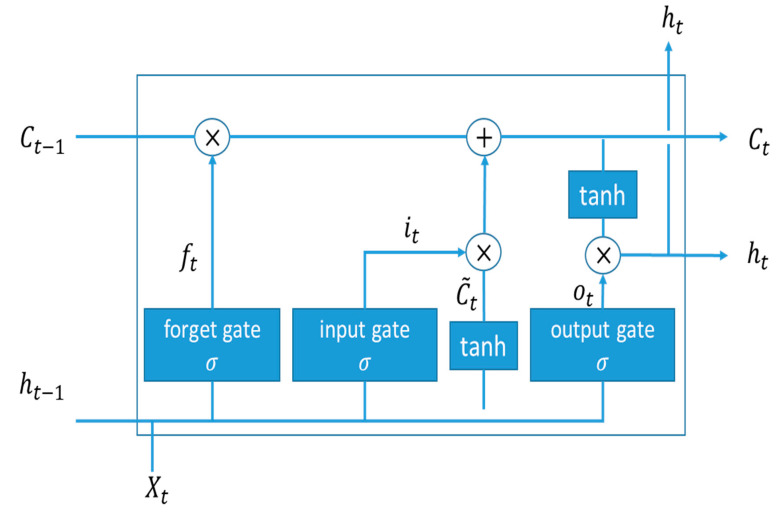
Basic structure of a long short-term memory (LSTM) model.

This subsection describes the learning models used in this work: the long short-term memory (LSTM) model and the multilayer perceptron (MLP) model. For the LSTM [21,22] model, it uses three gates to adjust previously stored data: input gate, output gate, and forgetting gate and improves the problem of the recurrent neural network (RNN) gradient vanishing. The forget gate is used to decide which information will be discarded from the cell state. The input gate determines how much new information is added to the cell state. The output gate is based on the cell state to determine what value is invited to output. The LSTM combines the structure of three gates to protect and control information. Therefore, the performance of LSTM is better than that of RNN in the task of long-term memory. In Figure 6, the upper horizontal line is the state of the cell. Selectively let messages through three gates. The forget gate is used to determine which messages pass through the cell, then enter the input gate, decide how many new messages to add to the cell state, and finally decide the output message through the output gate.

This work applies the LSTM with a forget gate. In Figure 6, $C_{t}$ and $h_{t}$ represent the cell state and the output value for the current moment, and $C_{t-1}$ and $h_{t-1}$ represent the cell state and the output value of the previous moment. Denote $f_{t}$, $i_{t}$, $o_{t}$, ${\tilde{C}}_{t}$ as forget gate’s activation vector, input/update gate’s activation vector, output gate’s activation vector, cell input activation vector, respectively. Let W and b be a weight matrix and a bias vector parameter, respectively, which need to be learned during training. Let $\;\sigma_{g}$ and $\;\sigma_{c}$ be the sigmoid function and the hyperbolic tangent (Tanh) function, respectively.

The first step is to decide what information to throw away from the cell state via a sigmoid layer called the forget gate layer. It looks at $h_{t-1}$ and input vector $x_{t}$, and outputs a number between 0 and 1 for each number in the cell state $C_{t-1}$. Note that a 1 represents “completely keep this” while a 0 represents “completely get rid of this”. Thus, the forget gate’s activation vector is given by

(1) $$f_{t}=\;\sigma_{g}\left(W_{f}\cdot \left[h_{t-1},x_{t}\right]\;+\;b_{f}\right)$$

The next step is to decide what new information to store in the cell state. The input gate layer and the Tanh layer are applied to create an update to the state.

(2) $$i_{t}=\;\sigma_{g}\left(W_{i}\cdot \left[h_{t-1},x_{t}\right]\;+\;b_{i}\right)$$

(3) $${\tilde{C}}_{t}=\;\sigma_{c}\left(W_{C}\cdot \left[h_{t-1},x_{t}\right]\;+\;b_{C}\right)$$

Then, the new cell state $C_{t}$ is updated by

(4) $$C_{t}=\;f_{t}*C_{t-1}+i_{t}*{\tilde{C}}_{t}$$

Finally, based on the cell state, we need to decide what to output. First, we run a sigmoid layer which decides what parts of the cell state for the output. Then, we put the cell state through and multiply it by the output of the sigmoid gate, which yields

(5) $$o_{t}=\;\sigma_{g}\left(W_{o}\cdot \left[h_{t-1},x_{t}\right]\;+\;b_{O}\right)$$

(6) $$h_{t}=\;o_{t}*\sigma_{c}\left(C_{t}\right)$$

An MLP [23] model consists of at least three layers of nodes (an input layer, a hidden layer, and an output layer). In the MLP model, some neurons use nonlinear activation functions to simulate the frequency of action potential, or firing of biological neurons. Since MLPs are fully connected, each node in one layer connects with a certain weight to every node in the following layer. After each data processing is completed, learning performs in the perceptron by adjusting the connection weights, which depends on the number of errors in the data output compared to the results.

The LSTM and MLP model architectures are paired with TensorFlow and Keras for model training. The LSTM parameter lookback is set to 5. The Adam optimization algorithm is applied for training the network. The loss value is evaluated via the root mean square error (RMSE). The activation functions use Tanh and scaled exponential linear units (Selu) functions. Units and activation functions of each layer are summarized in Table 5. Referring to the above LSTM layer, we can match the data by adjusting the number of cells, entering dimensions, and activating functions. The time distributed dense layer is to gradually apply the dense layer to the sequence. The dense layer is used to activate neurons in neural networks. For the MLP parameters, initialize weights with a normal distribution. The activation function uses the rectified linear units (Relu) function. Table 6 summarizes the units and activation functions at each layer of the MLP model, where the flatten layer is to flatten the high-dimensional matrix into a two-dimensional matrix, retaining the first dimension, and then multiplying the values of the remaining dimensions to get the second dimension of the matrix. When the model is trained, we can determine whether the model is overfitting such that the model can be adjusted according to the loss value and accuracy of each training. By testing the parameter values of different combinations and layers, the model suitable for the data is finally found.

The weather data collected by the sensors are processed according to the above data processing steps and format. The proposed model mainly focuses on predicting the weather condition for the coming day, including temperature, humidity, and air pressure. That is, assuming the current time is 0:00 with a weather prediction, the predicted temperature, humidity, and pressure values are obtained for the next 24 h (i.e., a weather prediction from 1:00 to 24:00). Accordingly, at 1:00 for performing an updated 24 h weather prediction (i.e., a weather prediction from 2:00 to the next day 1:00), the weather data collected by the sensors at 1:00 will be added to the original dataset to form a new input dataset for the sequential prediction of the next 24 h. Finally, the system accuracy is evaluated by the comparison between the predicted weather data and the measurement values via the root mean square error (RMSE), mean absolute error (MAE), and percentage error, as depicted in (1)–(3). Therefore, referring to the training model described above, weather prediction can be achieved.

(7) $$\mathrm{RMSE}\left(X,h\right)=\;\sqrt{\frac{1}{m}\overset{m}{\underset{i=1}{\sum }}{\left(h\left(x^{\left(i\right)}\right)-y^{\left(i\right)}\right)}^{2}}$$

(8) $$\mathrm{MAE}\left(X,h\right)=\frac{1}{m}\overset{m}{\underset{i=1}{\sum }}\left|h\left(x^{\left(i\right)}\right)-y^{\left(i\right)}\right|$$

(9) $$\mathrm{Percentage}\;\mathrm{Error}=\;\frac{\left|\mathrm{Predicted}\;\mathrm{value}-\;\mathrm{Exact}\;\mathrm{value}\right|}{\mathrm{Exact}\;\mathrm{value}}\;\times 100$$

The overall prediction model training process divides the original data into a training set, a verification set, and a test set after data processing, and then performs model training. After completing the model evaluation, the system adjusts the parameters according to the evaluation results and then continues training, and finally gets the prediction model.

After the data are processed, the trained prediction model is used to make a prediction. As the prediction is completed, the predicted values are added to the dataset to form a new dataset, and then the next prediction is performed until the final result is obtained, which completes the prediction task.

## 4. Experimental Results

To assess the system performance, this section explores information processing between bus and bus station and discusses the prediction performance of the learning models.

### 4.1. Information Processing Between Bus and Bus Station

**Figure 7:**
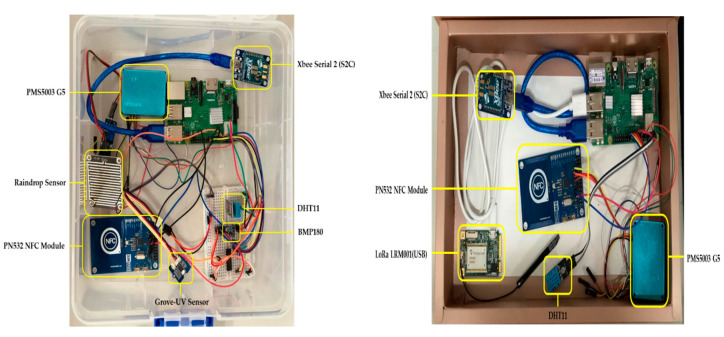
The bus experimental module (**left**); the bus station experimental module (**right**).

**Figure 8:**
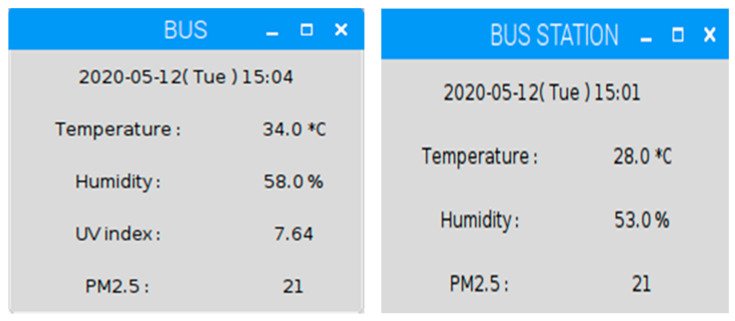
The weather information on the bus (**left**); environmental monitoring at the bus station (**right**).

Figure 7 (left) illustrates the bus experimental module, including sensors, a transceiver, a MCU board, and a NFC module. Figure 7 (right) depicts the bus station experimental module, including sensors, a transceiver, a MCU board, a NFC module, and a LoRa module. Figure 8 shows the weather information, updated every minute, on the bus and at the bus station, respectively.

**Figure 9:**
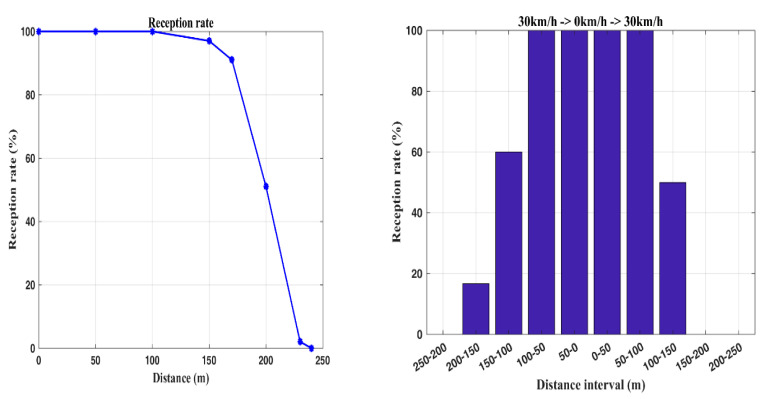
Reception rate at different distances (**left**); the reception rate of different distances when passengers get on and off: 30 km/h -> 0 km/h -> 30 km/h (**right**).

The following two experiments are executed to evaluate the stability of data reception, considering data transmission from a bus and data reception at a bus station with 1.5 s transmission time interval. The first experiment aims to determine the acceptable transmission range between a transmitter–receiver pair. Figure 9 (left) shows that the boundary of the transmission range with 97% data reception rate is about 150 m for a pair of static transmitters and receivers. Moreover, the data loss scenario happens with a transmission range of 200 m. For a farther transmission range, say a distance of 240 m, the reception rate drops to zero.

Due to the bus movement, the second experiment explores the impact of mobility on data reception performance. Based on the transmission range (about a distance of 240 m) from the first experiment, the data reception is examined where the bus moves towards the bus station upon arrival and departure. Under this circumstance, consider the passengers getting on or off the bus. Figure 9 (right) shows the data reception rates with a bus at the speed of 30 km/h in different communication ranges. Notice that the acceptable reception range is about 150 m with a 60% reception rate. In this work, we use an XBee module with an outdoor line-of-sight communication range up to 100 m. The reception variation for a distance above 100 m is because the signals reflected from various surrounding objects (multipath reception) converge at the receiving point. Therefore, possible obstacles (e.g., trees, the movement of people and vehicles) in an open space lead to a change in the quality of communication. At the same time, depending on the relative position of surrounding objects the signals can both be amplified and attenuated at the receiving point. Readers may refer to [24] for testing of communication range in ZigBee technology. In addition to exploring the reception rate, the results from Experiment 2 can be applied to determine the transmission time interval, considering the transmission range and the bus speed. For instance, with a bus at the speed of 30 km/h, the communication duration between the bus and the bus station is about 30 s, which suggests that the transmission time interval may be set for 10 s to ensure that the data can be received within the transmission range.

### 4.2. Prediction Performance

After the data are uploaded to the server’s database, predictions can be made through the trained prediction model. Referring to Table 4, two models are applied in this work: (1) the LSTM model and (2) the MLP model, considering the prediction of the temperature, humidity, and pressure values. The proposed models are evaluated on a large-scale database built by the EPA and CWB, Taiwan, which includes about 50,000 hourly measurements in the last six years of the Taichung Observatory (from 1 October 2013 to 10 June 2019). Note that the predicted values with the two models are compared with the actual measurements of the CWB on 21 March 2020 via RMSE, MAE, and percentage error.

#### 4.2.1. The LSTM Model

**Figure 10:**
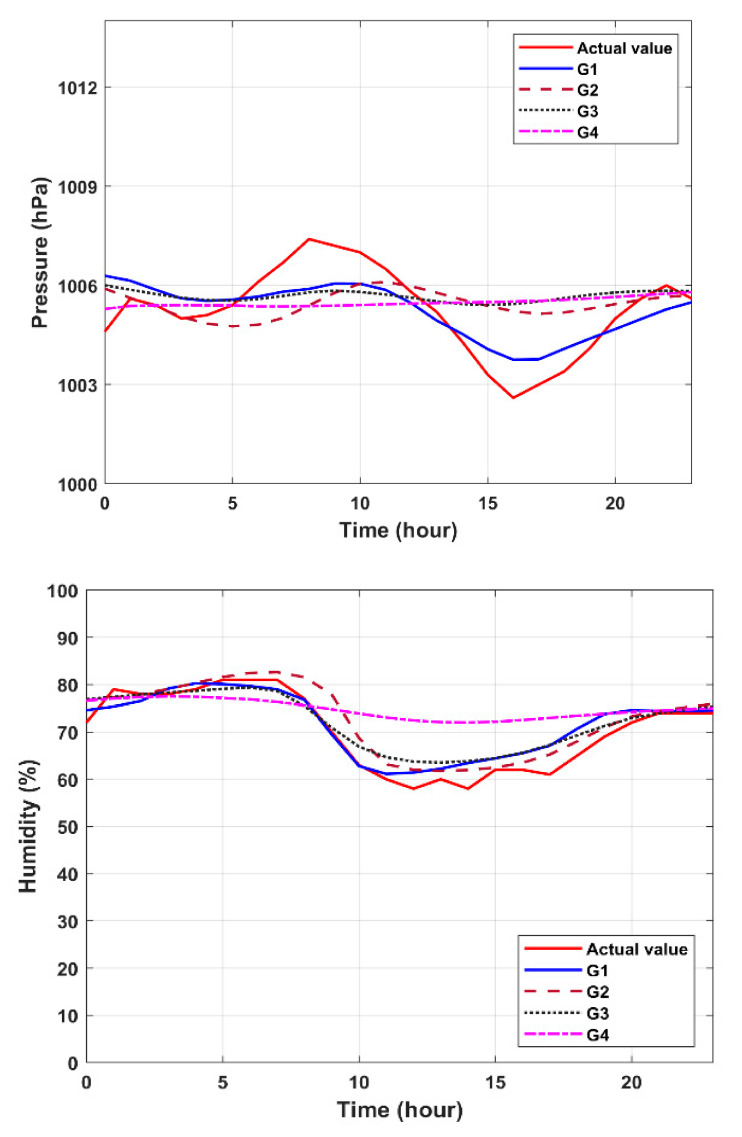

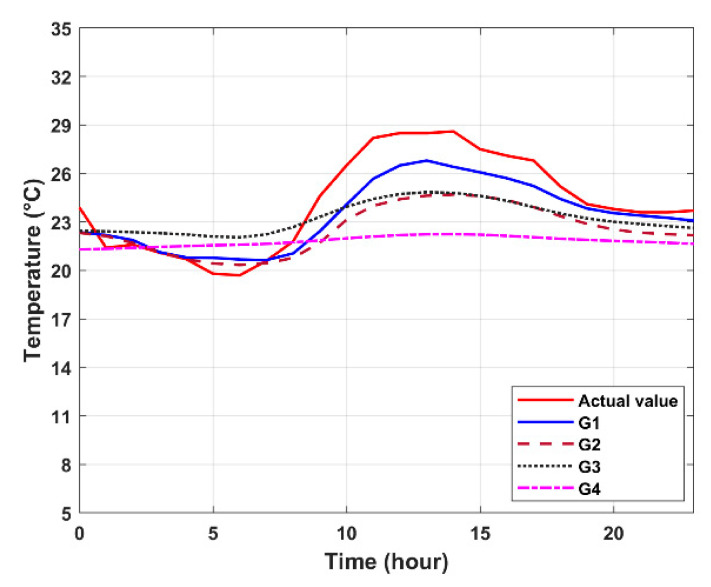
Forecast results of the LSTM model with the four input groups.

Referring to Table 4 for the LSTM model, Figure 10 is a prediction graph for the four input groups, with respect to temperature, humidity, and pressure. Table 7 shows the prediction results of different input data formats via RMSE and MAE values. Observe that the performance of the first input group (G1) is more in line with the actual values. Table 8 demonstrates the differences between the LSTM models with a different number of cells, calculating the loss value and accuracy of the test dataset, where the loss is calculated in mean square error, and the number of units used by the final model is fifty.

#### 4.2.2. The MLP Model

**Figure 11:**
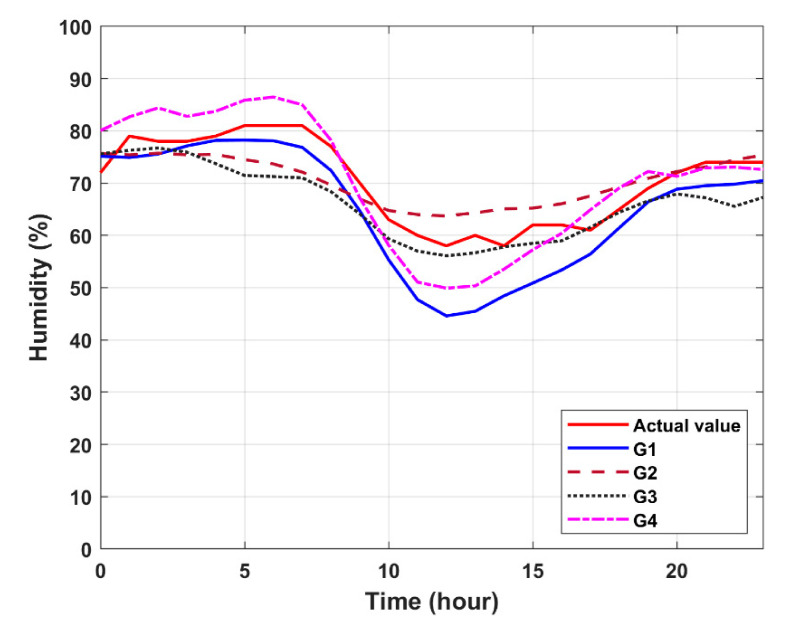

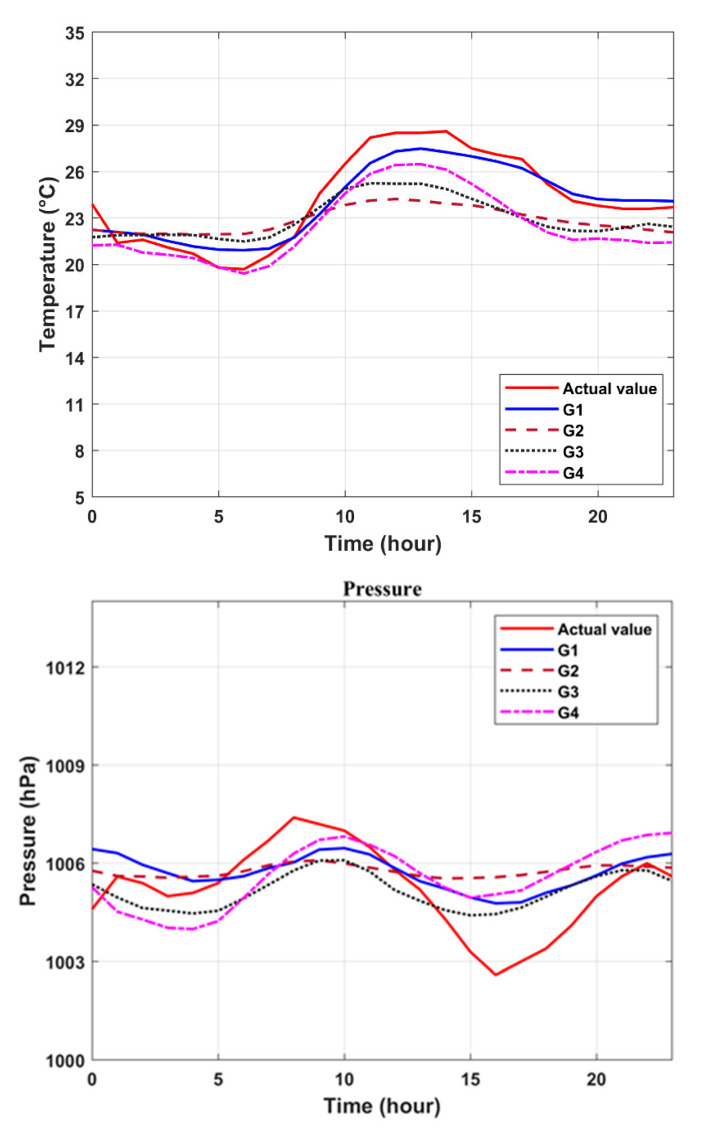
Forecast results of temperature, humidity, and pressure with the multilayer perceptron (MLP) model.

Given the input formats in Table 4 for the MLP model, Figure 11 is a prediction graph for the four input groups, from which the first input group (G1) of trends in the performances of temperature and air pressure are more in line with the actual values. Table 9 shows the prediction results via RMSE and MAE values. For the prediction of humidity, the prediction performance with the second input group (G2) has the smallest RMSE, MAE, and percentage error values (e.g., RMSE (G1) = 6.7972; RMSE (G2) = 0.4853; RMSE (G3) = 5.3816; RMSE (G4) = 4.9940). However, due to the lack of proper trend prediction as shown in Figure 11 and considering the overall prediction performances in temperature, humidity, and pressure, the first input group (G1) is selected to compare with the performance using the LSTM model.

**Figure 12:**
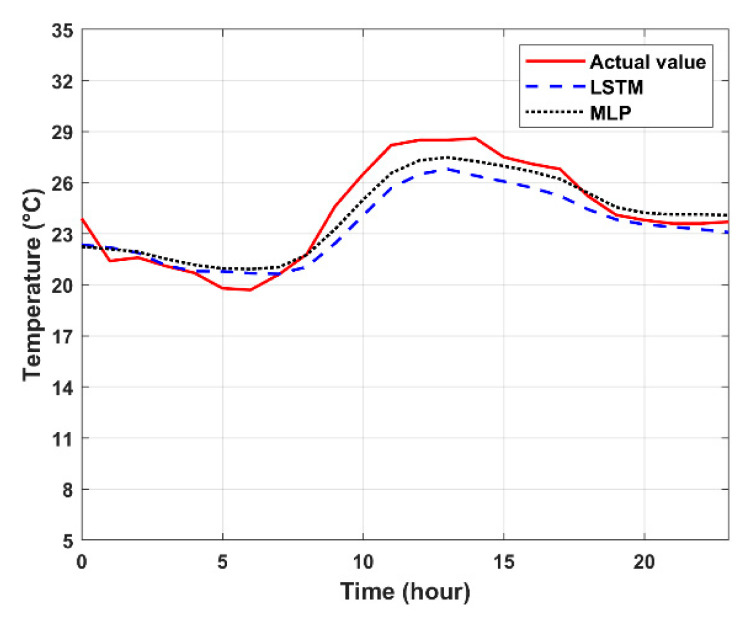

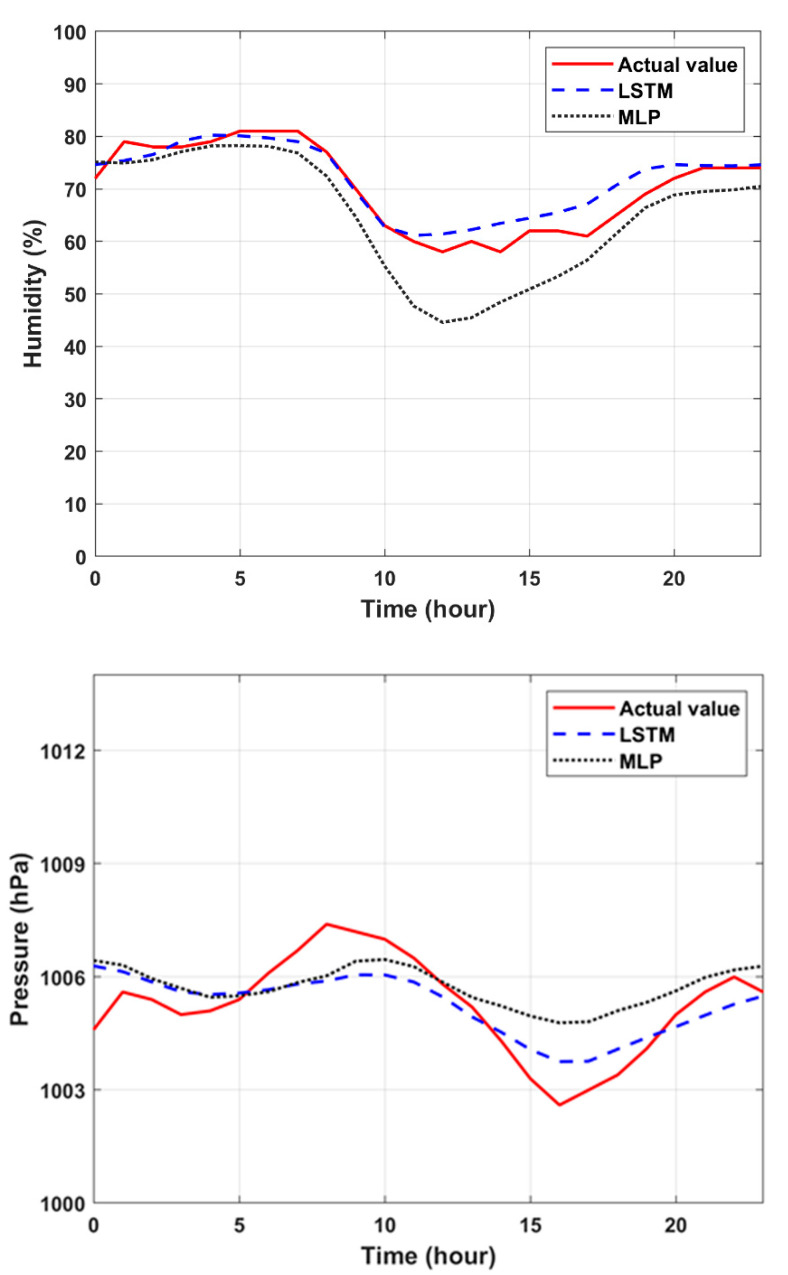
Forecast results of temperature, humidity, and pressure on 21 March 2020: LSTM model vs. MLP model.

With the input format G1, Figure 12 shows the comparison of prediction results of the LSTM and MLP models in terms of temperature, humidity, and pressure, which is summarized in Table 10. Observe that for the temperature prediction, the performances of the two models are close. From 8 a.m. to 6 p.m., the MLP performance is slightly better than the LSTM performance. However, the LSTM performance is better in other time periods. For the humidity and pressure parameters, the prediction performance of LSTM is superior to that of MLP for achieving a better tendency prediction. Although the LSTM shows a slight advantage over the MLP model, the above results may be due to the depth of the model at training or the differences in the number of cells and parameters used in each layer.

### 4.3. Comparison of Temperature Prediction

The proposed prediction system is compared with the system architectures in Parashar [14] and Varghese et al. [16]. In this work, the basic hardware architecture consists of the Raspberry Pi and the sensors. The data transmission and storage are carried out through wireless communication technologies and a database. Parashar [14] uses multiple linear regression (MLR) to train the model, which is a statistical technique that uses multiple explanatory variables to predict the outcome of the response variable. Then select the relative characteristic values of the highest, lowest, and average temperatures, respectively, for training. Finally, predict the highest, lowest, and average temperatures of the next day based on the weather data of the past three days. The system proposed in this paper uses the LSTM and MLP models for training and predicts the temperature, humidity, and pressure values within the next 24 h. Table 11 shows a comparison between the system of Parashar [14] and the proposed system, including training models, feature selections, prediction methods, and information display. The left side is the system of Parashar [14], while the right side is the proposed system.

The proposed system mainly predicts temperature, humidity. and pressure for the next 24 h. In contrast, the system in Parashar [14] only predicts the maximum, minimum, and average values of temperature of the day. In Varghese et al. [16], with Raspberry Pi and weather sensors, data are collected, trained, and predicted using linear regression (LR) machine learning models for evaluation via mean absolute error and median absolute error. The proposed prediction system is based on hourly weather data for training and prediction, and uses the percentage error, MAE, and RMSE as evaluation criteria. In addition to these three evaluation criteria, the explained variance of temperature is calculated for comparison with Parashar [14]. The comparison is mainly for the average temperature.

Table 12 describes the comparison of temperature prediction for the next day’s mean temperature. In terms of MAE performance, the LR model in Varghese et al. [16] has the MAE value, MAE (LR) = 2.5. The prediction performances of the system with the MLR model in Parashar [14] (MAE (MLP) = 1.10) and the proposed system with the LSTM model (MAE (LSTM) = 1.056) are close. And the proposed system with the MLP model has the lowest MAE value (MAE (MLP) = 0.7731), which suggests that the proposed models are good at prediction. In the explained variance, the performances of the proposed models are better than that of the literature as well. Moreover, the proposed system can provide past and current weather information and weather forecast values for the next 24 h.

To further assess the predictive skill, the predicted results of the proposed system are compared with the CWB actual values, the CWB predicted values, and those of the AccuWeather system [25], where the AccuWeather system is a Media Company in the United States, providing commercial weather forecasting services worldwide. Table 13 presents the comparison of actual values and the predicted results with respect to the highest and lowest temperatures on 21 March 2020 in Taichung. Observe that referring to the CWB forecast report [20], the highest and lowest temperatures of the CWB predicted values on 21 March 2020 in Taichung are in the 28–31 °C range and in the 18–21 °C range, respectively. For predicting the highest temperature, the AccuWeather system, the proposed LSTM model, and the proposed MLP model have 1.75%, 5.26%, and 3.51% prediction errors, respectively. For the prediction of the lowest temperature, the AccuWeather system, the proposed LSTM model, and the proposed MLP model have 11.11%, 2.53%, and 6.06% prediction errors, respectively, which suggest that the proposed system is competitive with the existing systems.

## 5. Conclusions

This paper presents a real-time weather monitoring and prediction system based on bus information management, combined with information processing and machine learning to complete the communication and analysis of information between buses, stations, and sensors. The proposed system contains four core components: (1) information management, (2) interactive bus stop, (3) machine learning prediction model, and (4) weather information platform. The website shows weather information via a dynamic chart. In addition to the current temperature, humidity, air pressure, rainfall, UV, and PM 2.5, the system provides a forecast of temperature, humidity, and air pressure for the next 24 h.

Although the proposed system achieves effective weather monitoring and information management, misalignment may be present due to the significant weather changes, which is the major challenge to overcome. In the future work, in addition to optimizing the system operation, we are planning to refine the prediction system, considering the deployment of nodes based on bus routes, the learning models, including more physical parameters, exploring the effects of forecast and measurement errors on the forecasting models, reanalyzing the dataset (e.g., performing data revisions), applying multiple data sources [26] and information processing technologies, which may achieve better prediction accuracy.

## Figures and Tables

**Table 1:** Comparison of prediction behaviors.

System	Training Model	Prediction
Parashar [14]	Multiple Linear Regression Model	Maximum and Minimum Temperatures on the Next Day Mean Temperature on the Next Day
Singh et al. [15]	The Random Forest Classification	Raindrop Prediction
Varghese et al. [16]	Linear Regression Model	Maximum and Minimum Temperatures on the Next Day
The Proposed System	Long Short-Term Memory Model Multilayer Perception Model	Temperature, Humidity, Pressure in the Next Twenty-Four Hours

**Table 2:** The sensor information.

Table Name	Time	Sensor Name			
Uv	Time	Ultraviolet	Bus stop		
Rf	Time	Rainfall	Bus stop		
Temp	Time	Temperature	Humidity	Bus stop	
Press	Time	Pressure	Bus stop		
Pm25	Time	Atpm10	Atpm25	Bus stop	
Pred_temp	Time	Temperature	Humidity	Pressure	Bus stop

**Table 3:** The unit of each sensor.

Name	Unit
Temperature	°C
Humidity	%
Ultraviolet	UV Index
Pressure	Pa
PM 2.5	μg/m^3^
Rainfall	0 à Rain 1 à No rain

**Table 4:** Input data format.

Group	Format of Data
G1	$\begin{array}{ccccc} \;In_{21}^{D-1} & In_{22}^{D-1} & In_{23}^{D-1} & In_{24}^{D-1} & In_{23}^{D} \end{array}$
G2	${\left[\begin{array}{ccccc} \;In_{21}^{D-1} & In_{22}^{D-1} & In_{23}^{D-1} & In_{24}^{D-1} & In_{23}^{D} \end{array}\right]}^{T}$
G3	${\left[\begin{array}{ccccc} \;In_{21}^{D-1} & In_{22}^{D-1} & In_{23}^{D-1} & In_{24}^{D-1} & In_{23}^{D} \end{array}\right]}^{T}$
G4	${\left[\begin{array}{ccccc} In_{24}^{D-3} & In_{1}^{D-2} & \ldots & In_{24}^{D-2} & \; \end{array}\begin{array}{ccc} In_{1}^{D-1} & \ldots & In_{24}^{D-1} \end{array}\begin{array}{ccc} \; & In_{1}^{D} & \ldots \end{array}\begin{array}{ccc} \; & In_{22}^{D} & In_{23}^{D} \end{array}\right]}^{T}$

**Table 5:** LSTM: units and activation functions of each layer.

Layer	Units	Activation Function
LSTM	50	tanh
Time Distributed Dense	30	
LSTM	30	tanh
Dense	15	selu
Dense	3	selu

**Table 6:** MLP: units and activation functions of each layer.

Layer	Units	Activation Function
Flatten	15	
Dense	15	relu
Dense	3	

**Table 7:** Forecast results of different input data formats with the LSTM model.

		G1	G2	G3	G4
RMSE	Temperature	1.3219	2.2876	2.21	3.5664
	Humidity	2.8696	3.1219	3.1839	7.6588
	Pressure	0.7676	1.2327	1.2753	1.338
MAE	Temperature	1.0561	1.8424	1.9387	2.8843
	Humidity	2.2483	2.4855	2.553	5.9607
	Pressure	0.6557	0.9508	1.0008	1.0208
Percentage Error	Temperature	4.15%	7.09%	7.79%	11.15%
	Humidity	3.39%	3.70%	3.93%	9.34%
	Pressure	0.07%	0.09%	0.10%	0.10%

**Table 8:** The loss value and accuracy of the LSTM model trained in different units.

Units	Test Loss	Test Acc
30	0.0638	0.913
40	0.0634	0.9118
45	0.0627	0.913
47	0.064	0.9106
48	0.0634	0.9117
49	0.0629	0.9114
50	0.0623	0.9125

**Table 9:** Forecast results of different input data formats with the MLP model.

		G1	G2	G3	G4
RMSE	Temperature	0.907	2.5569	2.2171	2.0243
	Humidity	6.7972	4.5853	5.3816	4.994
	Pressure	1.0369	1.2808	0.9948	1.271
MAE	Temperature	0.7731	2.2124	1.94	1.7937
	Humidity	5.604	3.9413	4.4632	4.3236
	Pressure	0.8433	0.965	0.8659	1.1227
Percentage Error	Temperature	3.17%	8.78%	7.73%	6.87%
	Humidity	8.61%	5.70%	6.11%	6.38%
	Pressure	0.08%	0.10%	0.09%	0.11%

**Table 10:** Forecast performance on 21 March 2020.

		Temperature	Humidity	Pressure
RMSE	LSTM	1.3219	2.8696	0.7676
	MLP	0.907	6.7972	1.0369
MAE	LSTM	1.0561	2.2483	0.6557
	MLP	0.7731	5.604	0.8433
Percentage Error	LSTM	4.15%	3.39%	0.07%
	MLP	3.17%	8.61%	0.08%

**Table 11:** Comparison table of training and prediction methods.

**Machine Learning**			
The system in Parashar [14]			The proposed system
**Training Model**			
2016.05.01 ~ 2018.03.11		2013.10.01 ~ 2019.06.10	
MLR		LSTM, MLP	
**Feature Selection**			
max temperature	Maxtempm_1×, Maxpressure_1×, Mintempm_3×, Maxpressure_3×, Meanpressurem_3		Temperature
min temperature	Mintempm_1×, Meantempm_3×, Maxtempm_1×, Maxtempm_2×, Maxdewptm_1×, Maxdewptm_3×, Meandewptm_1×, Meandewptm_2×, Meanpressure_1		Humidity
mean temperature	Meantemp_3×, Meanpressure_3×, Maxtemp_1×, Maxtemp_2×, Mintemp_1		Pressure
**Prediction**			
Maximum temperature, minimum temperature, mean temperature on the next day			Temperature, humidity, pressure in the next 24 h
Weather data for the past 3 days			The first type of data format (G1)
**Web Display**			
Present	Temperature, Min Temperature Max Temperature		Temperature, Humidity, Pressure, UV, PM25, Rain
Tomorrow	Min Temperature, Max Temperature Mean Temperature		Temperature, Humidity, Pressure

**Table 12:** Comparison table of the next day’s mean temperature (unit: °C).

Next Day Mean Temperature Accuracy				
	MLR [14]	LR [16]	LSTM	MLP
Explained Variance	0.95	N/A	0.865	0.904
Mean Absolute Error	1.10	2.5	1.0561	0.7731

**Table 13:** Comparison of the highest and lowest temperatures on 21 March 2020 (unit: °C).

Temperature					
	CWB Actual Value	CWB Predicted Value [20]	AccuWeather [25]	LSTM	MLP
Highest Temperature	28.5	28~31	29	27	27.5
Lowest Temperature	19.8	18~21	22	20.7	21
